# A Q Fever Outbreak on a Boer Goat Farm in South Korea: Molecular Typing and Serial Monitoring

**DOI:** 10.3390/ani16152303

**Published:** 2026-07-24

**Authors:** You-Jeong Lee, Jun Hyung Sohn, Beoul Kim, Hyo-Min Woo, Jae-Woo Choi, In-Soo Choi, Sehyun Son, Ok-Mi Jeong, Jin-Ju Lee, Yun Sang Cho, Dae Sung Yoo, Yong-Myung Kang, Dongmi Kwak, Jun-Gu Kang, Dongseob Tark, Gil Jae Cho, Min-Goo Seo

**Affiliations:** 1 College of Veterinary Medicine and Institute for Veterinary Biomedical Science, Kyungpook National University, Daegu 41566, Republic of Korea; wowgirlsgood@naver.com (Y.-J.L.); kbjjhnm@naver.com (B.K.); whmsm9902@naver.com (H.-M.W.); choiwoo777@naver.com (J.-W.C.); cis200232@naver.com (I.-S.C.); kamaboy88@knu.ac.kr (Y.-M.K.); dmkwak@knu.ac.kr (D.K.); 2 Gyeongbuk Veterinary Service Laboratory, Daegu 41566, Republic of Korea; vetsohn@korea.kr; 3 Bacterial Disease Division, Animal and Plant Quarantine Agency, Gimcheon 39660, Republic of Korea; scriptbar@korea.kr (S.S.); nabee@korea.kr (O.-M.J.); lejjj84@korea.kr (J.-J.L.); choys@korea.kr (Y.S.C.); 4 Department of Veterinary Epidemiology, College of Veterinary Medicine, Chonnam National University, Gwangju 61186, Republic of Korea; shanuar@jnu.ac.kr; 5 Laboratory for Infectious Disease Prevention, Korea Zoonosis Research Institute, Jeonbuk National University, Iksan 54531, Republic of Korea; herculess@jbnu.ac.kr (J.-G.K.); tarkds@jbnu.ac.kr (D.T.)

**Keywords:** *Coxiella burnetii*, goat, molecular typing, environmental surveillance, serial monitoring, One Health

## Abstract

Q fever is a zoonotic disease associated with reproductive losses in ruminants. This study investigated a 2025 outbreak on a Boer goat breeding farm in South Korea and compared its molecular characteristics with those of a separate 2024 outbreak. Conventional PCR targeting the IS1111 insertion element was used to test 87 goats, three female farm dogs, and four barn-level composite environmental samples. Positive goat samples underwent multiple-locus variable-number tandem-repeat analysis and multispacer sequence typing. *Coxiella burnetii* was detected in 60 of 87 goats (69.0%) and in one environmental sample, whereas dogs were negative. Two multiple-locus variable-number tandem-repeat profiles were identified among four goat samples, and two goats from different barns shared a profile provisionally designated MST77-like because it clustered near, but was not identical to, the MST77 reference profile. After movement restrictions, oxytetracycline treatment, environmental management, and serial monitoring, 11 of 57 previously positive goats remained positive at the second screening, and none of these 11 goats tested positive at the final examination. Complete herd clearance and the independent effect of antimicrobial treatment could not be confirmed because previously negative goats were not retested and no untreated comparison group was available. These findings support farm-wide testing, strengthened biosecurity, and follow-up monitoring.

## Simple Summary

Q fever is an infection that can cause abortion and stillbirth in goats and illness in people exposed to infected animals or contaminated farm materials. We investigated an outbreak on a Boer goat breeding farm in South Korea after reproductive losses and the death of an adult goat. The bacterium that causes Q fever was detected in 60 of 87 goats and in one of four pooled environmental samples, but not in three farm dogs. Genetic testing showed that the bacteria differed from those identified during a separate goat-farm outbreak investigated in 2024. After movement restrictions, antibiotic treatment, environmental management, and repeated testing, 11 of 57 previously positive goats remained positive at the second examination, and none of these 11 goats tested positive at the final examination. Because the entire herd was not retested, complete elimination could not be confirmed. These findings support early diagnosis, farm hygiene, follow-up testing, and cooperation between animal and public health services.

## 1. Introduction

*Coxiella burnetii* is an obligate intracellular bacterium and the causative agent of Q fever, an important zoonotic disease affecting humans and animals [1]. Domestic ruminants, particularly cattle, sheep, and goats, are the principal reservoirs. In pregnant ruminants, infection may be associated with abortion, stillbirth, premature delivery, and weak offspring [2,3,4]. Large numbers of organisms can be shed through placental tissues, birth fluids, vaginal secretions, milk, feces, and urine, leading to environmental contamination and aerosol exposure. Although cattle and sheep are also important reservoirs, goat-associated Q fever outbreaks are of particular public-health concern because infected pregnant goats may shed high concentrations of *C. burnetii* in placental membranes, birth fluids, and abortion products during parturition or abortion, thereby increasing environmental contamination and the risk of aerosol transmission to humans [5,6,7,8,9]. Goat-farm outbreaks have been associated with abortion storms, prolonged bacterial shedding, contaminated dust and farm environments, and human Q fever cases among exposed workers, visitors, animal purchasers, and nearby residents [5,6,7,8]. Therefore, Q fever outbreaks on goat farms have implications for animal production, occupational health, and public health.

In South Korea, serological and molecular studies have shown that *C. burnetii* circulates in several domestic and wild animal populations, including cattle, goats, horses, dogs, and wild boars [10,11,12,13,14]. Goat-associated Q fever is particularly relevant because infected herds may experience reproductive losses and may also pose exposure risks to humans. A large outbreak reported on a goat farm in Cheongju in 2018 involved a high infection rate among goats and multiple human cases associated with exposure to the affected farm [15]. Population-based studies have also detected *C. burnetii* in Korean native goats and Boer goats, indicating ongoing circulation in domestic goat populations [13,16]. In addition, a previous investigation of a Boer goat farm outbreak in Gyeongbuk Province in 2024 identified animal and environmental infection and characterized the detected strains using multiple-locus variable-number tandem-repeat analysis and multispacer sequence typing [17].

The present Brief Report describes another independent Q fever outbreak that occurred in 2025 on a Boer goat breeding farm in a different locality of Gyeongbuk Province, South Korea. Following abortion and stillbirth events, farm-wide screening was conducted in goats, farm dogs, and barn-level composite environmental samples, and serial follow-up testing was performed after outbreak-control measures. Selected PCR-positive goat samples were further characterized by molecular typing and compared with profiles from the separate 2024 outbreak and public reference databases. This study aimed to summarize the epidemiological features, molecular typing results, and follow-up PCR findings of this focused field outbreak investigation.

## 2. Materials and Methods

### 2.1. Ethical Approval

All animal-related procedures were conducted in accordance with the protocol approved by the Institutional Animal Care and Use Committee of Kyungpook National University on 26 June 2024 (approval no. KNU 2024-0281). Fecal and environmental samples were collected using non-invasive procedures, whereas vaginal swabs and tissue specimens were collected by licensed veterinarians. All animal handling and sampling procedures were performed in accordance with applicable institutional and national guidelines for animal welfare. Permission for animal sampling and the use of farm-related information was obtained from the farm owner. No human diagnostic testing, biological specimen collection, or medical-record review was conducted as part of this study.

### 2.2. Case History and Sample Collection

In October 2025, a commercial Boer goat breeding farm located in Seongju, Gyeongbuk Province, Republic of Korea, housed 87 goats and three female farm dogs. The farm regularly sold goats to other farms. Goats were maintained in four modified vinyl greenhouse-type barns in shared pens on soil floors covered with rice-straw bedding. Controlled access points, quarantine areas, vehicle disinfection facilities, and mechanical ventilation systems were not available (Figure S1).

On 28 October 2025, a diagnostic investigation was requested following the death of an adult goat and the occurrence of abortion and stillbirth cases on the farm. Tissue specimens were collected from one deceased adult goat and three stillborn fetuses and submitted for differential diagnostic testing. PCR testing detected *C. burnetii* in all four animals, and the Q fever outbreak was officially confirmed on 4 November 2025.

On 10 November 2025, a first-round expanded screening was conducted following confirmation of the outbreak. Vaginal swabs were collected from female goats, whereas fecal samples were collected from male goats. Vaginal swabs were also obtained from the three female farm dogs. In addition, four composite environmental samples, one representing each barn, were collected. For each barn, dust, soil, and fecal material were collected from multiple locations within and around the farm facilities, including the farm entrance, areas surrounding fences, manure-composting areas, kidding areas, feed-storage areas, pillars, fences, floors, walls, feed bins, ventilation fans, facility entrances, and door handles. Specimens collected from multiple locations associated with each barn were pooled to produce one composite environmental sample per barn. All animal and environmental samples were examined by PCR for *C. burnetii*.

For molecular characterization, four PCR-positive goat samples were selected for multiple-locus variable-number tandem-repeat analysis (MLVA), and two PCR-positive goat samples were selected for multispacer sequence typing (MST). Samples were selected on the basis of strong PCR amplification and sufficient DNA quality and quantity for reliable multilocus amplification and sequence analysis.

Following the first-round screening, oxytetracycline treatment was administered under the direction of the attending veterinarian as part of the outbreak-control measures. Oxytetracycline (Terra-10 Inj., 100 mg/mL oxytetracycline HCl; manufactured by P&B Animal Health, Hwaseong, Republic of Korea) was administered intramuscularly at 10–15 mg/kg body weight once daily for five consecutive days from 14 to 18 November 2025. This regimen was selected based on veterinary clinical judgment, the formulation used on the farm, and published caprine oxytetracycline use and pharmacokinetic data, including systemic administration at 10 mg/kg and pharmacokinetic evaluation of conventional and long-acting formulations at 20 mg/kg in goats [18,19]. An additional dose at the same dosage was administered on 3 December 2025.

On 4 December 2025, a second-round screening was conducted. Of the 60 goats that had tested positive during the first-round screening, 57 were available for follow-up testing because three goats had died before the second examination. The three female farm dogs were also resampled during the second-round screening. Four composite environmental samples, representing the same four barns, were collected again and examined for *C. burnetii*. Following the second-round screening, additional oxytetracycline treatment was administered under veterinary supervision to goats that remained PCR-positive. Oxytetracycline was administered intramuscularly at 10–15 mg/kg body weight once daily from 15 to 18 December, from 22 to 24 December, and from 26 to 28 December 2025.

On 29 December 2025, a third-round screening was conducted on the 11 goats that had remained PCR-positive during the second-round examination. Because Q fever is a zoonotic disease, the farm owner and spouse were informed of the potential public health risks and advised to seek medical evaluation. During a subsequent epidemiological interview, the farm owner reported that both individuals had received positive Q fever test results at a medical institution; this information was self-reported and was not independently verified by the investigators.

### 2.3. DNA Extraction

Total DNA was extracted using commercial kits selected according to specimen type. Tissue specimens and vaginal swabs were processed using the DNeasy *Blood & Tissue* Kit (Qiagen, Melbourne, Australia) in accordance with the manufacturer’s instructions. Fecal samples collected from goats, as well as environmental soil and fecal-material specimens, were processed using the Beniprep Soil/Fecal DNA Extraction Kit (InVIRUStech, Gwangju, Republic of Korea). Environmental dust specimens were processed using the Clear-S™ Quick DNA Extraction Kit (InVIRUStech). Dust, soil, and fecal-material specimens were extracted separately using the corresponding extraction kits, and the resulting DNA extracts were pooled by barn to generate four composite environmental DNA samples.

### 2.4. Screening for Abortion-Related Diseases

To investigate potential infectious causes of the reproductive losses observed on the farm, tissue samples collected from one deceased adult goat and three stillborn fetuses were subjected to differential molecular testing for 13 pathogens associated with abortion and reproductive disorders in ruminants.

Real-time PCR assays were performed using commercially available PowerChek™ Real-time PCR kits (Kogenebiotech, Seoul, Republic of Korea) to detect *Toxoplasma gondii*, *Listeria monocytogenes*, *Campylobacter fetus*, *Salmonella Dublin*, and *Brucella abortus*, in accordance with the manufacturer’s instructions. Multiplex reverse-transcription PCR assays were performed for Akabane virus and Aino virus using the VDx^®^ Bovine Akabane/Aino MP RT-PCR Kit (Median Diagnostics, Chuncheon, Republic of Korea), and for Chuzan virus, bovine ephemeral fever virus, and Ibaraki virus using the VDx^®^ Bovine Chuzan/BEF/Ibaraki MP RT-PCR Kit (Median Diagnostics). Conventional PCR assays were performed to detect *Neospora caninum* and *Leptospira* spp. using the LiliF™ Neospora PCR Kit and LiliF™ LEPTO PCR Kit, respectively (iNtRON Biotechnology, Seongnam, Republic of Korea). *C. burnetii* was detected by conventional PCR targeting the IS1111 insertion element, as described in Section 2.5.

### 2.5. Conventional PCR Detection of Coxiella burnetii

Conventional PCR targeting the multicopy IS1111 insertion element of *C. burnetii* was performed using AccuPower^®^ PCR PreMix (Bioneer, Daejeon, Republic of Korea). Amplification was conducted using the previously described Trans-1 and Trans-2 primers [20], which generate an expected amplicon of 687 bp. DNA extracted from a previously characterized *C. burnetii*-positive goat sample from South Korea was used as the positive control [17]. A no-template control was included in each PCR run to monitor potential contamination.

### 2.6. MLVA Genotyping

MLVA was performed on four *C. burnetii*-positive goat samples. The samples were selected on the basis of strong IS1111 PCR amplification and sufficient DNA quality and quantity for multilocus analysis. Six variable-number tandem-repeat loci—Ms23, Ms24, Ms27, Ms28, Ms33, and Ms34—were amplified individually using AccuPower^®^ PCR PreMix (Bioneer, Daejeon, Republic of Korea) and the corresponding primer sets, following previously described procedures [21]. The resulting allelic profiles were expressed in the order Ms23–Ms24–Ms27–Ms28–Ms33–Ms34 and compared with reference profiles available in the *C. burnetii* MLVA database (version 3.1), accessed on 3 February 2026.

### 2.7. MST Genotyping

MST was performed on two *C. burnetii*-positive goat samples selected on the basis of strong IS1111 PCR amplification and sufficient DNA quality for complete multilocus analysis. Ten intergenic spacer regions—Cox2, Cox5, Cox18, Cox20, Cox22, Cox37, Cox51, Cox56, Cox57, and Cox61—were amplified individually using AccuPower^®^ PCR PreMix (Bioneer, Daejeon, Republic of Korea) and the corresponding primer sets, following previously described procedures [21]. The nucleotide sequence obtained for each spacer was assigned an allele number by comparison with reference sequences, and the resulting allelic profiles were arranged in the order Cox2–Cox5–Cox18–Cox20–Cox22–Cox37–Cox51–Cox56–Cox57–Cox61. The composite spacer profiles were compared with reference profiles available in the *C. burnetii* MST database, accessed on 3 February 2026. Profiles showing the closest genetic similarity to, but not complete identity with, a registered MST genotype were designated as MST-like profiles.

### 2.8. DNA Sequencing and Phylogenetic Analysis

Selected IS1111 amplicons, PCR products from the six MLVA variable-number tandem-repeat loci, and PCR products from the ten MST spacer regions were submitted to Macrogen (Seoul, Republic of Korea) for Sanger sequencing using the corresponding amplification primers. For analysis of the IS1111 region, 16 PCR-positive goat samples were selected on the basis of amplicon quality and suitability for sequencing. MLVA amplicons obtained from four selected goat samples were sequenced to determine the repeat numbers and allelic profiles at each locus. MST amplicons obtained from two selected goat samples were sequenced to determine the allele sequence of each spacer region.

Sequence chromatograms were manually inspected, trimmed, and edited using BioEdit v7.2.5. The IS1111 sequences were aligned with reference sequences retrieved from the NCBI GenBank database using CLUSTAL Omega v1.2.1, and nucleotide sequence identities were evaluated by pairwise comparison. For MST-based phylogenetic analysis, the nucleotide sequences of the ten spacer regions were concatenated in the order Cox2–Cox5–Cox18–Cox20–Cox22–Cox37–Cox51–Cox56–Cox57–Cox61 and aligned with representative reference MST sequences. Maximum-likelihood phylogenetic trees were constructed using MEGA v6.0. Evolutionary distances were estimated using the Kimura two-parameter substitution model, and the robustness of the inferred tree topologies was assessed by bootstrap analysis with 1000 replicates.

### 2.9. Statistical Analysis

PCR results were summarized descriptively as numbers and percentages of positive samples. No inferential statistical analyses were performed.

## 3. Results

### 3.1. Detection of Abortion-Related Pathogens

Specimens collected from one deceased adult goat and three stillborn fetuses were subjected to differential molecular testing for 13 pathogens associated with abortion and reproductive disorders in ruminants. Among the pathogens examined, only *C. burnetii* was detected. Conventional PCR detected *C. burnetii* in specimens from all four animals, whereas the other 12 pathogens were not detected.

### 3.2. Serial Conventional PCR Detection of Coxiella burnetii

During the first-round screening conducted on 10 November 2025, *C. burnetii* was detected in 60 of 87 goats (69.0%), with barn-level positivity ranging from 19.0% in Barn 1 to 100.0% in Barn 2 (Table 1). All three female farm dogs tested negative. Of the four barn-level composite environmental samples, only the sample representing Barn 3 tested positive.

During the second-round screening conducted on 4 December 2025, 57 of the 60 initially positive goats were available for follow-up testing because three goats had died before re-examination. Among these 57 goats, 11 (19.3%) remained PCR-positive and 46 tested negative. Ten of the 11 remaining positive goats were housed in Barn 2, and one was housed in Barn 3. All four composite environmental samples and all three female farm dogs tested negative during the second-round screening.

During the third-round screening conducted on 29 December 2025, the 11 goats that had remained PCR-positive during the second-round screening were re-examined. None of these 11 goats tested positive for *C. burnetii* by conventional PCR.

### 3.3. MLVA Profiles of Coxiella burnetii

MLVA genotyping was successfully performed on four *C. burnetii*-positive goat samples selected on the basis of strong IS1111 PCR amplification. Analysis of the six VNTR loci—Ms23, Ms24, Ms27, Ms28, Ms33, and Ms34—identified two allelic profiles. Goats No. 16 and No. 137 exhibited the MLVA profile 9-27-4-6-9-5. Comparison with reference profiles in the *C. burnetii* MLVA database showed similarity to profiles previously reported from tick- and human-derived samples in the United States, human blood and vascular samples in France, human vascular samples in Canada, and bovine milk samples in Poland. Similar reference profiles had previously associated with MST16. Goats No. 8 and No. 17 exhibited the MLVA profile 1-10-2-3-7-3, which was most similar to the profile 20090317Frankrijk065 identified from a human plasma sample in France and previously associated with MST12.

### 3.4. MST Genotyping and Phylogenetic Analysis

**Figure 1:**
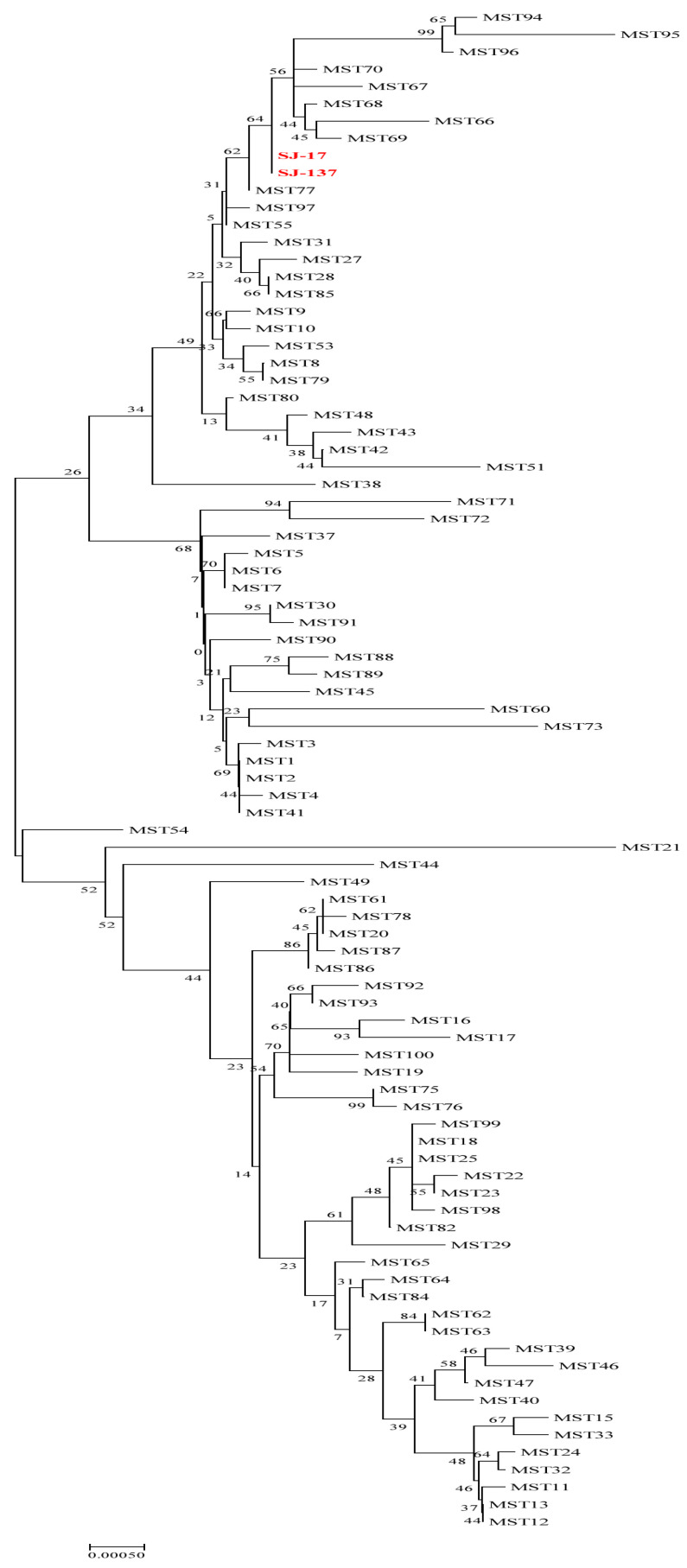
Maximum-likelihood phylogenetic tree based on the concatenated sequences of ten multispacer sequence typing loci of *Coxiella burnetii*. The MST77-like profile identified in goats No. 17 and No. 137 is indicated in red. The two sequences clustered near, but were not identical to, the registered MST77 reference profile. Reference profiles were retrieved from publicly available databases. The tree was constructed in MEGA v6.0 using the Kimura two-parameter model with 1000 bootstrap replicates.

MST genotyping was successfully completed for two *C. burnetii*-positive goat samples collected from different barns. Goats No. 17 and No. 137 exhibited an identical allelic profile of 5-4-9-5-1-5-14-16-4-6 across the Cox2, Cox5, Cox18, Cox20, Cox22, Cox37, Cox51, Cox56, Cox57, and Cox61 spacer regions. Comparison with reference profiles in the *C. burnetii* MST database indicated that this profile was closely related to, but not identical with, the registered MST77 profile. Therefore, because the profile has not been assigned a formal MST number in the database, it was provisionally designated an MST77-like. In the MST-based maximum-likelihood tree, the sequences obtained from goats No. 17 and No. 137 clustered near the MST77 reference profile, supporting their close genetic relationship with MST77 rather than complete identity (Figure 1).

### 3.5. IS1111 Sequence Analysis

Sixteen representative goat samples were selected from the 60 PCR-positive samples for sequencing of the IS1111 amplicon. The 16 partial IS1111 sequences shared 99.1–100% pairwise nucleotide identity with one another and 98.8–100% identity with previously reported *C. burnetii* reference sequences from South Korea, China, and Germany, including sequences derived from goat, human, and tick samples (Figure S2). The sequences generated in this study were deposited in GenBank under accession numbers PZ123603–PZ123618.

## 4. Discussion

This Brief Report describes an independent Q fever outbreak associated with abortion and stillbirth on a Boer goat breeding farm in South Korea. Farm-wide screening detected *C. burnetii* in 60 of 87 goats (69.0%), with marked variation among barns, and one of four barn-level composite environmental samples was PCR-positive. The goat-level PCR positivity rate was comparable to that reported during the 2018 Cheongju goat-farm outbreak, in which 54 of 77 goats (70.1%) were PCR-positive and 63 of 77 (81.8%) were seropositive [15], but was higher than the rates reported in broader population-based surveys of Korean native goats and Boer goats [13,16]. This difference likely reflects the outbreak-based nature of the present investigation, which was initiated following abortion, stillbirth, and mortality events, rather than routine population surveillance.

The timing of the outbreak should be considered in relation to reproductive events rather than calendar season alone. Q fever outbreaks in goats may show an apparent seasonal pattern corresponding to the kidding period because infected pregnant goats can shed large quantities of *C. burnetii* through placental tissues, birth fluids, and vaginal secretions during abortion or parturition [22,23]. In the present investigation, detection of *C. burnetii* coincided with abortion and stillbirth events during October and November. Thus, the observed timing may have been associated with reproductive activity and increased periparturient bacterial shedding. However, because long-term surveillance was not conducted, this study cannot establish a seasonal pattern for the farm.

The source and route of introduction of *C. burnetii* could not be determined because detailed animal-movement and epidemiological tracing data were unavailable. The farm regularly sold breeding goats and lacked quarantine facilities, controlled entry points, and vehicle disinfection facilities. These conditions may have allowed an infection already present in the herd to persist or may have created opportunities for introduction and dissemination through animal, personnel, vehicle, or equipment movements. Management characteristics such as larger herd size, higher stocking density, and intensive production practices have previously been associated with herd-level *C. burnetii* positivity on commercial goat farms [24]. Nevertheless, these explanations remain hypotheses, and no particular introduction route or management factor could be causally linked to the present outbreak.

Only the composite environmental sample representing Barn 3 tested positive, although all 40 goats in Barn 2 were PCR-positive during the first-round screening. This discordance should not be interpreted as evidence that environmental contamination was absent or unimportant. Environmental detection of *C. burnetii* may be affected by the timing and location of sampling, the type of matrix examined, heterogeneous distribution of contaminated material, and the analytical sensitivity of the assay [5,25,26]. In the present study, samples collected from multiple locations were pooled to produce a single composite environmental DNA sample for each barn. Although this approach provided a broad barn-level assessment, pooling may have diluted localized or low-level contamination and prevented identification of specific contaminated sites. Individual analysis of dust, soil, bedding, surfaces, and abortion-associated areas would have provided greater spatial resolution. In addition, environmental specimens were not consistently collected from identical locations during each sampling round, limiting direct longitudinal comparison. Environmental matrices may also contain substances that interfere with DNA extraction or PCR amplification. Because internal amplification controls, inhibitor-removal assessments, and quantitative bacterial-load measurements were not included, low-level contamination and PCR inhibition could not be distinguished from true negative results. The negative environmental findings should therefore be interpreted cautiously.

During the epidemiological interview, the farm owner reported that both the owner and spouse had received positive Q fever test results at a medical institution. These self-reported findings were not independently verified because the investigators did not examine medical records, collect human biological specimens, or perform human diagnostic testing. Furthermore, no human-derived sequences were available for comparison with the goat-derived sequences; therefore, direct animal-to-human transmission during this outbreak could not be demonstrated. Nevertheless, goat-associated outbreaks in other countries have shown that infected breeding herds may pose risks to farm residents, workers, visitors, animal purchasers, and surrounding communities [7,8]. The present findings reinforce the importance of personal protective equipment, hand and respiratory hygiene, safe handling and disposal of placental and fetal materials, prompt medical evaluation of exposed persons, and coordinated veterinary and public-health responses.

MLVA identified two profiles among four selected goat samples. The profile 9-27-4-6-9-5 was similar to reference profiles reported from tick- and human-derived samples in the United States, human blood and vascular samples in France, human vascular samples in Canada, and bovine milk samples in Poland; comparable database profiles had previously been associated with MST16. The profile 1-10-2-3-7-3 was most similar to profile 20090317Frankrijk065, which was obtained from a human plasma sample in France and had previously been associated with MST12. These similarities indicate that related molecular profiles have been detected in different host species and geographic regions, but they do not demonstrate direct epidemiological connections or international transmission.

MST analysis showed that goats No. 17 and No. 137, which originated from different barns, shared an identical study profile of 5-4-9-5-1-5-14-16-4-6. In the phylogenetic analysis, their concatenated spacer sequences clustered close to, but were not identical with, the registered MST77 reference profile. The study profile was therefore provisionally described as MST77-like rather than MST77. Because formal assignment of a new MST type requires database comparison, validation, and registration, it was not designated as a novel MST genotype in the present study. The MST77-like profile differed from the MST55 and MST80 genotypes detected during the separate Boer goat farm outbreak investigated in 2024 [17], suggesting molecular variation among outbreaks occurring at different farms and times in South Korea. Goats No. 17 and No. 137 also exhibited different MLVA profiles despite sharing the same MST77-like profile. This is not necessarily contradictory because MLVA and MST examine different genomic regions and have different discriminatory capacities; multiple MLVA profiles may occur within a smaller number of MST groups [27,28]. However, because MLVA and MST were successfully completed for only four and two samples, respectively, the detected profiles may not represent the full diversity of *C. burnetii* present on the farm, particularly if mixed infections occurred.

The partial IS1111 sequences obtained from 16 selected goat samples showed high nucleotide identity with one another and with reference sequences from South Korea, China, and Germany. These findings support molecular confirmation of *C. burnetii* in the outbreak samples. However, IS1111 is a multicopy insertion element used primarily as a sensitive diagnostic target and has limited discriminatory power for fine-scale epidemiological inference. Clustering with human-, goat-, or tick-derived reference sequences should therefore not be interpreted as evidence of a common source or direct transmission. Higher resolution methods, including expanded multilocus typing, single-nucleotide-polymorphism analysis, or whole-genome sequencing, would be required to determine relationships among outbreak-associated strains [29].

Following implementation of movement restrictions, environmental management, oxytetracycline treatment, and serial monitoring, the number of PCR-positive goats decreased from 60 during the first-round screening to 11 of 57 goats during the second-round screening. None of these 11 goats tested positive at the final examination. Nevertheless, this temporal pattern does not establish the efficacy of oxytetracycline. *C. burnetii* is an intracellular pathogen with marked tropism for placental trophoblasts, and bacterial shedding in goats is strongly associated with pregnancy, abortion, parturition, and the timing of specimen collection [22,25]. High levels of bacterial DNA may be detected around parturition and subsequently decline, and shedding may also be intermittent or differ among vaginal, fecal, milk, and reproductive specimens. Consequently, the final negative PCR results may have reflected the combined effects of the natural course of infection, changes in reproductive status, temporal variation in shedding, specimen type, management measures, and repeated antimicrobial treatment.

Evidence that oxytetracycline reduces abortion or *C. burnetii* shedding in infected goat herds remains limited, and well-controlled goat field trials are lacking [30]. Studies in small ruminants have reported inconsistent effects of oxytetracycline-based interventions on bacterial shedding [11,31]. Moreover, no untreated comparison group was available in the present investigation, and treatment was administered together with several other control measures. The independent contribution of antimicrobial therapy to the final molecular results therefore could not be determined. Conventional PCR also detects bacterial DNA and cannot distinguish viable organisms from nonviable organisms or residual DNA.

Complete herd clearance could not be confirmed because goats that tested negative during earlier screening rounds were not all retested at the final examination. Confirmation of true herd clearance would have required final testing of all remaining animals in the herd, potentially together with repeated environmental testing. Additional limitations included the use of different specimen types according to goat sex, unavailable individual demographic and reproductive data, the lack of standardized environmental sampling locations, possible environmental PCR inhibition, and the small number of samples suitable for molecular typing. Future investigations should repeatedly test both initially positive and negative animals, apply consistent specimen-collection protocols, analyze environmental samples individually, include controls for PCR inhibition, and integrate higher resolution genomic analysis with detailed animal-movement and verified human epidemiological information.

## 5. Conclusions

This study documented a Q fever outbreak on a Boer goat breeding farm in South Korea, with *C. burnetii* detected in 60 of 87 goats and in one barn-level composite environmental sample. Molecular typing identified two MLVA profiles and an MST77-like profile that differed from the genotypes detected during a separate goat-farm outbreak investigated in 2024. However, because MLVA and MST were completed for only four and two selected samples, respectively, the detected profiles may not represent the full genetic diversity of *C. burnetii* on the farm, particularly if mixed infections occurred. PCR positivity decreased during serial monitoring after the implementation of antimicrobial treatment and other outbreak-control measures, but the independent effect of oxytetracycline could not be determined. Complete herd clearance also could not be confirmed because goats that had previously tested negative were not retested during the final round; confirmation would require final testing of all animals. These findings emphasize the importance of farm-wide diagnosis, strengthened biosecurity, safe management of reproductive materials, and comprehensive follow-up monitoring during goat-associated Q fever outbreaks.

## Figures and Tables

**Table 1:** Serial conventional PCR results for *Coxiella burnetii* during the 2025 outbreak investigation.

Screening Round	Specimen Group	Barn	No. Tested	No. Positive (%)
First, 10 November 2025	Goats	1	21	4 (19.0)
		2	40	40 (100)
		3	15	10 (66.7)
		4	11	6 (54.5)
	Goat subtotal	-	87	60 (69.0)
	Composite environmental samples	1–4	4	1 (25.0) ^1^
	Dogs	-	3	0
Second, 4 December 2025	Goats	1	4	0
		2	40	10 (25.0)
		3	7	1 (14.3)
		4	6	0
	Goat subtotal	-	57	11 (19.3)
	Composite environmental samples	1–4	4	0
	Dogs	-	3	0
Third, 29 December 2025	Goats	2	10	0
		3	1	0
	Goat subtotal	-	11	0

^1^ Only the composite environmental sample representing Barn 3 tested positive. Three of the 60 initially positive goats died before the second-round screening. The third-round screening included only goats that remained positive during the second-round screening.

## Data Availability

Data supporting the conclusions of this article are included within the article. The newly generated sequences were submitted to the GenBank database under the accession numbers PZ123603–PZ123618. The datasets used and/or analyzed during the present study are available from the corresponding author upon reasonable request.

## Supplementary Materials

The following supporting information can be downloaded at https://www.mdpi.com/article/10.3390/ani16152303/s1, Figure S1: Housing conditions at the Boer goat breeding farm affected by Q fever in 2025. (A) Interior and (B) exterior views of the modified greenhouse-type barns. Goats were housed in shared pens on soil floors covered with rice-straw bedding. Controlled entry, quarantine, vehicle disinfection, and mechanical ventilation facilities were absent.; Figure S2: Maximum-likelihood phylogenetic tree based on partial IS1111 insertion-element sequences of *Coxiella burnetii.* Sixteen sequences obtained from PCR-positive goat samples in the present study are shown in blue, and reference sequences retrieved from GenBank are labeled with their accession numbers. The tree was constructed in MEGA v6.0 using the Kimura two-parameter model with 1000 bootstrap replicates.
